# Dimeric and Multimeric DNA Aptamers for Highly Effective Protein Recognition

**DOI:** 10.3390/molecules25225227

**Published:** 2020-11-10

**Authors:** Claudia Riccardi, Ettore Napolitano, Domenica Musumeci, Daniela Montesarchio

**Affiliations:** 1 Department of Chemical Sciences, University of Naples Federico II, via Cintia 21, I-80126 Naples, Italy; ettore.napolitano@unina.it (E.N.); domenica.musumeci@unina.it (D.M.); daniela.montesarchio@unina.it (D.M.); 2 Department of Advanced Medical and Surgical Sciences, 2nd Division of Neurology, Center for Rare Diseases and InterUniversity Center for Research in Neurosciences, University of Campania Luigi Vanvitelli, via Sergio Pansini, 5, I-80131 Naples, Italy; 3 Institute of Biostructures and Bioimages, CNR, via Mezzocannone 16, I-80134 Naples, Italy

**Keywords:** aptamer, G-quadruplex, design, dimerization, multivalency, molecular recognition, protein target, therapy

## Abstract

Multivalent interactions frequently occur in biological systems and typically provide higher binding affinity and selectivity in target recognition than when only monovalent interactions are operative. Thus, taking inspiration by nature, bivalent or multivalent nucleic acid aptamers recognizing a specific biological target have been extensively studied in the last decades. Indeed, oligonucleotide-based aptamers are suitable building blocks for the development of highly efficient multivalent systems since they can be easily modified and assembled exploiting proper connecting linkers of different nature. Thus, substantial research efforts have been put in the construction of dimeric/multimeric versions of effective aptamers with various degrees of success in target binding affinity or therapeutic activity enhancement. The present review summarizes recent advances in the design and development of dimeric and multimeric DNA-based aptamers, including those forming G-quadruplex (G4) structures, recognizing different key proteins in relevant pathological processes. Most of the designed constructs have shown improved performance in terms of binding affinity or therapeutic activity as anti-inflammatory, antiviral, anticoagulant, and anticancer agents and their number is certainly bound to grow in the next future.

## 1. Introduction

Nucleic acid-based aptamers are short single-stranded DNA or RNA molecules which, upon folding in their peculiar three-dimensional structure, can bind with high affinity and specificity a selected target of biological interest. They are also called “chemical antibodies”, but compared to protein-based molecules, oligonucleotide aptamers generally show lower immunogenicity, higher stability in a wide range of pH and temperature and the possibility to be easier modified or conjugated. Indeed, site-specific chemical modifications can be easily inserted in oligonucleotide aptamers to improve their stability to nuclease digestion or modulate binding affinity to their target [1,2,3,4,5,6]. These intriguing properties make oligonucleotide aptamers very attractive tools in both therapeutic [2,7,8,9,10,11,12,13,14,15] and diagnostic [16,17,18,19,20,21,22] applications [23].

Starting from a large pool of random oligonucleotide sequences, high affinity aptamers for a given target are generally identified through an in vitro selection process named Systematic Evolution of Ligands by Exponential Enrichment (SELEX) [24,25]. The outstanding progress achieved in this field resulted in a variety of selection methods and a large number of aptamers specific for very different kinds of targets—from small molecules, ions, proteins, cells, to even whole organisms, such as viruses or bacteria—have been thus far fished out [26,27,28,29,30,31,32].

Moreover, several aptamers are specifically internalized upon binding to cell membrane receptors and thus can serve as ideal selective delivery systems for different therapeutic targets, from small, conventional drugs to microRNAs or small interfering RNAs (siRNAs) [10,33,34].

Notably, among combinatorially selected aptamers, most of the oligonucleotides endowed with valuable biological activity are able to adopt stem-loop or G-quadruplex (G4) structures. The simplest architecture is represented by the stem-loop or hairpin rearrangement, i.e., an intramolecular conformation based on the coupling of complementary nucleobases in a single-stranded DNA sequence [35].

In contrast, oligonucleotides featured by guanine-rich sequences generally share the ability to fold into peculiar G4 structures [36,37,38,39,40]. The central core of a G4 architecture is the G-tetrad, a structural motif also named G-quartet, which consists of a cyclic planar arrangement of four guanine bases associated through Hoogsteen-type hydrogen bonds [38,40,41,42,43]. Stacking of two or more G-tetrads provides central cavities with a strong negative electrostatic potential, in which cations can be well accommodated, strongly influencing the formation, stability, and topology of the resulting G4 structure [44,45,46,47].

Considering that protein targets involved in specific diseases can (i) have more than one potential binding site recognized by different aptamers, (ii) be dimeric, tetrameric, or in general multimeric, (iii) dimerize or multimerize as a consequence of physiological or pathological events, multivalent aptamer constructs, especially in the simple dimeric forms, are of particular interest [48,49,50,51]. Specifically, a multivalent aptamer is a construct composed of two or more units of the same or different aptamer motifs, containing or not additional structural elements or functional linkers, able to interact simultaneously with more protein binding sites, generally improving its overall efficacy.

Remarkably, SELEX often identifies oligonucleotide aptamers with a repeated sequence, suggesting high affinity recognition ability by dimeric aptamers for a given protein. As an alternative, since aptamers are largely amenable to chemical modifications [1,2,3,4,5,6], the oligonucleotide sequences initially discovered by SELEX can be easily modified to give dimeric or multimeric aptamers without linkers or using proper spacers of different nature (nucleotidic or not), length and flexibility, and exploiting different kinds of connecting interactions (base-pairs recognitions, covalent chemical linkages). Therefore, oligonucleotide aptamers represent a rich arsenal of finely tunable building blocks, which can be profitably joined to generate suitable constructs with improved functions and properties.

Most exploited strategies involve the simple combination of two of more aptamer units concurrently binding two different domains of a target protein with key biological functions in physiological and pathological conditions.

This review is focused on dimeric and multimeric DNA-based aptamers as therapeutic tools targeting key proteins in different relevant diseases, such as inflammation, viral infection, thrombosis and cancer. We here describe the selection, design and properties of multivalent DNA-based aptamers in terms of binding affinity and therapeutic efficacy.

## 2. Anti-Inflammatory Aptamers

L-, E-, and P-selectins are calcium-dependent cell surface lectins that mediate leukocyte extravasation from the vasculature into surrounding tissues [52]. All three selectins share a similar structure with an amino-terminal calcium-dependent lectin domain, an epidermal growth factor-like domain and complement binding-like domains [53]. In particular, L-selectin (CD62L) is constitutively expressed on the surface of most circulating leukocytes [54], while the expression of E- and P-selectins on endothelial cells and/or platelets can be induced [52].

Having the ability to modulate leukocyte extravasation—a hallmark of inflammation and homeostatic trafficking—L-selectin plays a key role in inflammation and injury [55,56,57]. Therefore, it is considered a fundamental therapeutic target in inflammatory diseases, stimulating the research of suitable aptamers designed to specifically target and inhibit its function [58].

In the mid-1990s, Hicke and coworkers first identified three L-selectin binding DNA aptamers through SELEX procedures. These 39-mer oligodeoxyribonucleotides (ODNs)—named LD201, LD174, and LD196—proved to be specific for L-selectin over P- and E-species, showing similar binding affinity, in the nanomolar range, to the lectin domain of L-selectin (Table 1). These aptamers were also able to inhibit lymphocyte adhesion and trafficking in vitro and in vivo [59].

**Figure 1:**
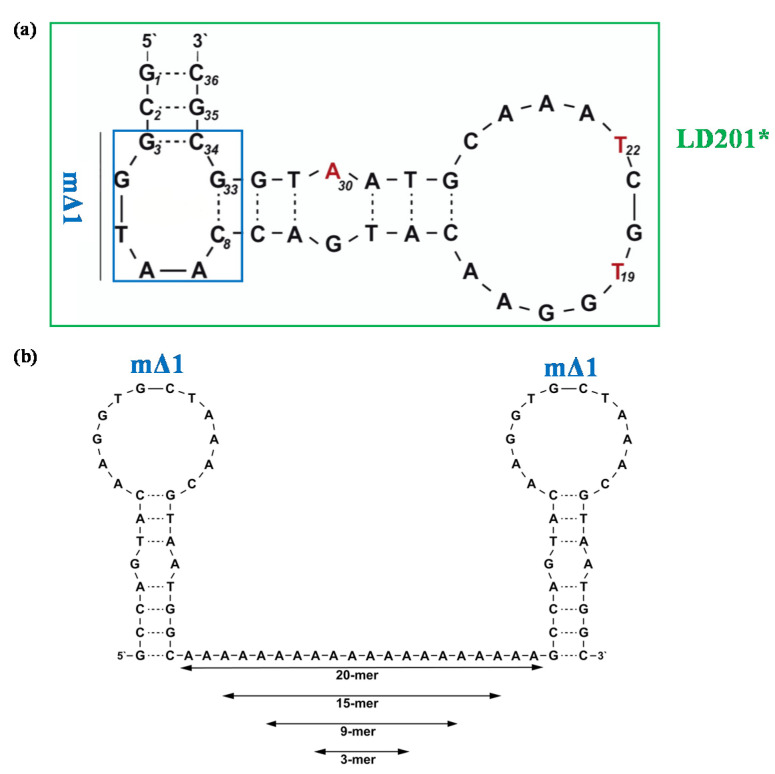
Schematic representation of the secondary structure of the L-selectin targeting aptamers. (**a**) Structure of the monomeric aptamers LD201* and mΔ1, as predicted by the mfold software for nucleic acid folding prediction [64]. The blue box in LD201* sequence represents the nucleotides removed in the truncated mΔ1 analogue (G3-C8 and G33-C34); (**b**) structure of the mΔ1 dimer in which two aptamer units are linked with poly(dA) linkers of different length. Figures were adapted from Riese et al. [61] with permission.

A slightly modified and shorter version of the LD201 aptamer (i.e., LD201*, Figure 1a and Table 1) was then specifically designed for an efficient affinity-based purification of L-selectin [60].

Subsequently, Riese and colleagues evolved a truncated form of LD201*, i.e., mΔ1, devoid of 8 bases (G3-C8 and G33-C34, Figure 1a) with respect to the starting sequence. The obtained 28-mer oligonucleotide showed IC_50_ values comparable to LD201* along with improved thermal stability, expressed in terms of melting temperature values (T_m_, Table 1) [61]. Therefore, mΔ1 was selected as a starting building block for the construction of dimeric and trimeric constructs [61]. Specifically, two mΔ1 units were linked via flexible poly(dA) spacers of different length (i.e., containing 3, 9, 15, and 20 deoxyadenosines; Figure 1b), obtaining dΔ1-A3, dΔ1-A9, dΔ1-A15, and dΔ1-A20, respectively [61]. All these dimers were tested for their inhibitory activity on L-selectin by means of competitive surface plasmon resonance (SPR) measurements using well-known L-selectin binders as competitive ligands [61]. Compared to the parent mΔ1, dΔ1-A3 dimer exhibited higher IC_50_ values, suggesting that the length of the exploited linker was too short and thus detrimental for the aptamer activity. On the contrary, improved inhibitory activity was found for the other three dimers, with the best results obtained for dΔ1-A9 showing the lowest IC_50_ value in the series (0.3 nM, Table 1) [61].

Stimulated by these intriguing findings, the authors also prepared a trimeric oligonucleotide in which 3 mΔ1 units were connected one to each other through a 9-mer poly(dA) spacers, providing tΔ1-A9. Compared to its monomeric counterpart, this trimeric construct exhibited improved inhibitory activity (IC_50_ = 0.8 nM, Table 1) which was however lower than that of the dΔ1-A9 dimer, probably for steric hindrance effects [61]. Both the dimer and trimer with (dA)_9_ spacers demonstrated leukocyte blocking capability in vitro and the trivalent aptamer also efficiently inhibited leukocyte rolling in model mice [61].

In a different design, in order to improve the selective and high affinity targeting of L-selectin, Chang and colleagues generated multivalent forms of the LD201 aptamer [62] using circular DNA templates and exploiting the rolling circle amplification (RCA) method. RCA is a simple and powerful isothermal enzymatic reaction in which a DNA polymerase extends the DNA sequence from a primer by replicating a circular DNA template many times to yield a single stranded(ss) DNA product [63].

Chang et al. used a circular template corresponding to the complementary sequence of the L-selectin aptamer and designed aptamer units connected by a 20 nucleotide poly(T) sequence in the RCA product, reflecting the poly(dA) tract in the circle template. The resulting long, linear ssDNA product (termed LS-Multi-Aptamer) incorporated ca. 30 consecutive copies of the L-selectin aptamer [62]. LS-Multi-Aptamer exhibited approximately a 10^3^ higher affinity for L-selectin than the monovalent aptamer, as evaluated by flow cytometry. Additionally, it showed IC_50_ values of ~0.75 nM on Jurkat cells, showing a sensibly improved activity than the corresponding monomer counterpart (>1 μM). Finally, LS-Multi-Aptamer proved to inhibit at nanomolar concentrations the interaction between L-selectin and endogenous ligands on the cell surface and block cell homing in secondary lymphoid tissues in mice [62].

## 3. Antiviral Aptamers

In the context of antiviral therapy, several aptamers have been identified against key proteins of the viral lifecycle. These aptamers can inhibit virus binding and entry into the target cell or virus integration, thus resulting as potent and specific antiviral agents [36,40,65,66,67]. 

**Figure 2:**
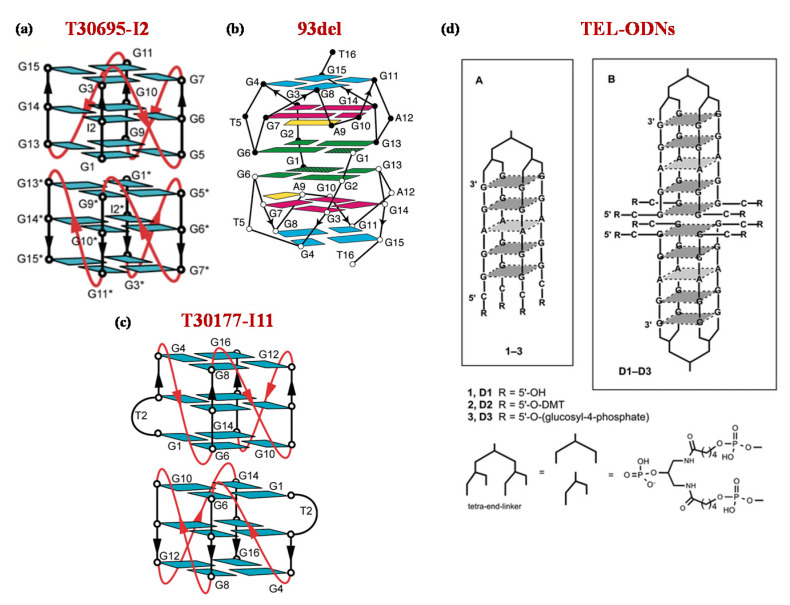
Schematic representation of the G-quadruplex folding topology adopted by T30695-I2 (**a**), 93del (**b**) and T30177-I11 (**c**) in K^+^-solutions as proposed by Do et al. [75], Phan et al. [76] and Mukundan et al. [77], respectively. For both T30695 and T30177, NMR spectra were obtained from derivatives including a single guanine-to-inosine substitution at position 2 or 11, respectively. (**d**) Schematic representation of the TEL-ODNs as monomers (**A**) and dimers (**B**). Figures were reproduced from refs [75,76,77,78] with permission.

Notably, among the known anti-human immunodeficiency virus type 1 (HIV-1) aptamers, many of them proved to spontaneously form dimeric G4 structures in solutions, such as the aptamers 93del, T30177 and T30695 targeting HIV-1 integrase (Figure 2) [36,65].

In detail, 93del is the 16-mer oligonucleotide of sequence 5′-GGGGTGGGAGGAGGGT-3′, found to inhibit HIV-1 integrase—an enzyme that allows the integration of the HIV genetic material into the DNA of the infected cell—with an IC_50_ value of 42 nM [68,69,70].

The 17-mer T30177 aptamer (5′-G*TGGTGGGTGGGTGGG*T-3′, where * indicates phosphorothioate internucleoside linkages) showed effective inhibition of HIV-1 integrase with IC_50_ of ca. 100 nM [71,72].

In the search for optimized T30177 analogues, the 16-mer T30695, with sequence 5′-G*GGTGGGTGGGTGGG*T-3′, was designed and found to have T_m_ values substantially higher than its precursor, as well as comparable inhibitory activity on HIV-1 replication in cell culture [73,74]. 

From a structural point of view, T30695 forms a dimeric G4 structure stabilized by stacking of two propeller-type parallel-stranded G4 units, in which all the guanine nucleobases are involved in the G-tetrad core formation (Figure 2a) [75].

In turn, as revealed by in-depth nuclear magnetic resonance (NMR) studies, 93del is able to adopt a dimeric interlocked parallel-stranded G4 architecture (Figure 2b) [76], while T30177 folds into a dimeric G4 structure, with six G-tetrad layers involving the stacking of two propeller-type parallel-stranded G4s at their 5′-end (Figure 2c). All guanine residues of the sequence participate in the G-quartet formation, with an interruption in the first G-tract due to a thymidine residue forming a bulge between two adjacent G-tetrads [77].

In parallel with the discovery of aptamers able to spontaneously adopt a dimeric structure in solution, some dimeric constructs were also ad hoc designed to exploit multivalency effects [78].

Nici et al. developed three different tetra-end-linked (TEL) dimers (1–3) containing four arms of the sequence 5′-CGGAGG-3′ attached through the 3′-end to a tetra-branched and differing for the groups at the 5′ position. Indeed, TEL-ODNs were provided with 5′-hydroxyl groups uncapped (1) or capped with either the lipophilic 4,4′-dimethoxytrityl (DMT) (2) or the hydrophilic glucosyl-4-phosphate (pGlc) (3) moieties (Figure 2d) [78]. The 5′-CGGAGG-3′ sequence was specifically selected since it includes the 5′-CGGA-3′ unit allowing for 5′–5′ end-stacking dimerization [79,80] and presents a GG-TEL moiety, previously exploited in one of the most active anti-HIV aptamers [81] targeting the HIV envelope glycoprotein 120 (gp120), essential for viral entry [36].

Among the designed dimers, only the DMT-substituted TEL-ODN 2 proved to be effective in protecting human MT-4 cell cultures from HIV infection (Table 1), suggesting that the presence of a terminal bulky, hydrophobic moiety can favor the binding with a specific pocket of the target glycoprotein [78], in line with previous data acquired on 5′-modified derivatives of the G4-forming aptamer 5′-TGGGAG-3′ [82].

## 4. Anticoagulant Aptamers

The most popular protein target for anticoagulant therapies is thrombin, a multifunctional “trypsin-like” serine protease able to bind fibrinogen and thus catalyze its conversion to fibrin clots in the last step of blood coagulation [83,84,85,86,87,88].

Considering its pivotal role in the coagulation cascade, the inhibition of thrombin activity is one of the most efficient antithrombotic strategies [89,90,91,92]. In this context, the development of effective antithrombin aptamers has been the focus of several investigations [92,93,94,95,96]. 

The most popular antithrombin aptamers able to inhibit thrombin activity are TBA15, TBA29, and TBA27 [92,96]. Besides differing for their overall length, these aptamers show distinct three-dimensional structures and recognize different thrombin binding sites [96].

Indeed, TBA29 and TBA27—also known as HD22-29 and HD22-27—adopt a mixed duplex/G4 architecture able to bind the exosite II of thrombin (heparin-binding site or ABE II) with high affinity (*K*_d_ values of 0.5 and 0.7 nM, respectively for TBA29 and TBA27) [97,98]. In particular, TBA27 is a truncated form of TBA29 (5′-AGTCCGTGGTAGGGCAGGTTGGGGTGACT-3′) lacking the first and last residue of the parent aptamer. In contrast, TBA15 or simply TBA, of sequence 5′-GGTTGGTGTGGTTGG-3′, folds into a stable chair-like, antiparallel G4 structure able to inhibit the conversion of soluble fibrinogen into insoluble fibrin strands by binding to thrombin exosite I (fibrinogen-binding site or ABE I) with a *K*_d_ of 26 nM [99,100,101,102,103,104,105,106].

Starting from these G-rich oligomers, several homo and heterodimeric constructs were developed as effective antithrombin agents [48,96].

**Figure 3:**
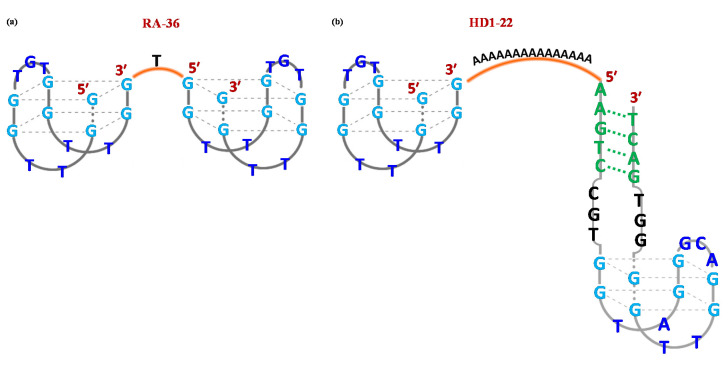
Schematic representation of the G-quadruplex structure formed in solution by the homodimeric RA-36 (**a**) and heterodimeric HD1-22 (**b**) thrombin-targeting aptamers. Figures were redrawn from Amato et al. [109] and Müller et al. [110] respectively.

RA-36 is the simplest TBA-based homodimeric aptamer. This 31-mer oligonucleotide comprises two TBA15 units, both in 5′→3′ direction, covalently linked through one thymidine residue at position 16 (Figure 3a). As its monomeric precursor, RA-36 recognizes thrombin exosite I, inhibiting the binding between the protein and fibrinogen [107]. Notably, this dimer was able to exert its activity only in a K^+^-rich solution, suggesting that the formation of a stable G4 structure is strictly required for effective inhibition of thrombin catalytic action [107,108].

The intriguing properties of RA-36 stimulated the design of other dimeric TBA15 variants, obtained by joining the 3′-ends of each G4 module and introducing inversion of polarity sites in the overall sequence. The connection between TBA15 motifs was realized using various symmetric linkers—i.e., deoxyadenosine or thymidine residues and/or a glycerol moiety—in place of the thymidine at position 16. Unfortunately, the direct comparison of the anticoagulant properties of the newly developed derivatives with the parent RA-36 was not performed, but the evaluation of prothrombin times revealed improved anticoagulant activity and higher T_m_ values for most of the designed dimers, compared to unmodified TBA15 (Table 1) [109].

Alternative dimeric constructs were based on the covalent connection between TBA15 and other antithrombin aptamers providing heterodimers able to recognize different thrombin exosites.

In particular, TBA15 and TBA29 were linked exploiting different spacers, such as a 15-nt long poly(dA) linker providing HD1-22 (Figure 3b) [110,111] or poly(T) spacers of various length [112]. 

In all cases, TBA15/TBA29 dimeric derivatives showed improved thrombin affinity and/or inhibition activity with respect to each monovalent parent aptamer, especially when a poly(T) spacer of 5 residues was explored (Table 1) [110,111,112].

In a different approach, the same aptamers were joined by ethylene glycol spacers of different length. For instance, Tian and Heyduk prepared a covalent dimer of TBA15 and TBA29 featured by flexible connections based on 5′-(OCH_2_CH_2_)_6_-OPO_3_-3′ (spacer 18) repeated 5 or 10 times (Table 1) [113]. Five repeated units of the spacer provided an overall linker length of 12 nm, while 10 repetitions allowed reaching a 24 nm-long spacer. This longer version was used both to link the 3′-end of TBA29 with the 5′-end of TBA15 and vice versa. In all cases, the designed bivalent analogues proved to be more efficient in terms of thrombin binding affinity than the starting monomeric aptamers (Table 1) [113].

In turn, Hughes et al. inserted an inverted thymidine (^i^T) at the 3′-end position of TBA15 and TBA29, thus providing RNV216A and RNV219, respectively [114]. These modified versions were then linked by using either a triethylene glycol (TEG) spacer (RNV220) or four thymidine residues (RNV220-T). Compared to both monovalent aptamers, RNV220 and RNV220-T showed significantly improved antithrombin activity in blood plasma (Table 1) [114].

As a valuable alternative to rational design, Ahmad and coworkers used an in vitro selection strategy to identify the optimal sequence joining TBA15 and TBA29 motifs. The randomized linker was 35-nt long, covering the distance between the different thrombin binding exosites [115]. The resulting 119-mer bivalent aptamer (TBV-08) exhibited noteworthy thrombin binding affinity in the picomolar range (*K*_d_ of 8.1 pM, Table 1) which well correlated with improved antithrombin activity. Similarly to previous approaches, the authors also prepared bivalent constructs presenting poly(T) or poly(dA) linkers. Interesting results in terms of *K*_d_ values were also found for the dimer containing a poly(T) spacer of 16 residues (Table 1) [115].

Only one study reported the covalent connection of TBA15 and the shorter HD22 aptamer, i.e., TBA27. In detail, from 2 to 10 units of the commercially available hexaethylene glycol (HEG)-based phosphoramidite (indicated as S, Table 1) were inserted to join the different monomeric aptamer units. The best results in terms of thrombin inhibitory activity were found for the analogue including 8 repeated units of the linker phosphoramidite 18 (Table 1), corresponding to a length of ca. 16 nm [116]. On the contrary, introduction of the shortest spacer of the series, i.e., the one constituted by 4 repeated units of phosporamidite 18, dramatically reduced the inhibitory activity of the obtained dimer analogue [116].

In order to simultaneously increase the resistance to nuclease degradation and the thrombin binding properties, Di Giusto and colleagues proposed circular multivalent constructs [117,118]. Indeed, circularization is a well-established strategy to improve the performance of aptamers [119,120] and has been efficiently applied to antithrombin aptamers targeting thrombin exosite I [96,121,122,123].

In this work, four different DNA aptamers were exploited as building blocks to obtain circular multivalent constructs: antithrombin aptamers TBA29 and GS-522 (i.e., a 15-mer with the sequence 5′-GGTTGGTGAGGTTGG-3′ able to bind thrombin exosite I [99] ), the L-selectin aptamer [59] and the aptamer against red blood cell marker. A DNA hairpin loop was included as an ancillary module between the aptamer motifs and all the resulting oligonucleotides were also elongated with flanking regions to allow extended stem-loop structure formation. Intra- or intermolecular DNA ligation approaches were used to provide each multivalent circular species, which finally involved two, three or four identical or different aptamer units [117]. The circular multivalent aptamers showed noteworthy stability in both serum and plasma along with improved anticoagulant activity compared to each parent antithrombin aptamer [117].

Another strategy to generate homo- or heteromultimers of a selected aptamer is based on the use of suitable nanoplatforms on which multiple copies of the monomeric aptamer can be linked [48]. This approach has been extensively investigated for TBA15, which thanks to its short sequence and well-known three-dimensional structure, well conserved also when bound to thrombin [124,125], is often exploited as a model system in proof-of-concept studies [48].

In this context, TBA15 was incorporated onto very different nanoplatforms, including magnetic [126,127,128], gold [129,130,131,132,133], silica-based nanoparticles [134,135,136,137], and graphene [138,139].

As a remarkable example, Hsu et al. proposed the multimerization of both TBA15 and TBA29 on the surface of gold nanoparticles (AuNPs). To reach this aim, both aptamers were equipped with thiol end groups allowing their attachment onto the gold surface through Au-thiol interactions [131]. The resulting multivalent nanoparticles, functionalized with about 15 molecules of each aptamer, exhibited an exceptionally high binding affinity for thrombin with a *K*_d_ value of 3.4 fM [131].

## 5. Anticancer Aptamers

DNA aptamers have demonstrated promising potential in biomedicine, especially in cancer therapy, targeting different protein targets [12,140,141,142,143].

### 5.1. Receptor Tyrosine Kinase PTK7

Receptor tyrosine kinase PTK7 is a cancer-specific cell surface marker overexpressed in several subtypes of leukemia, including T-cell acute lymphoblastic leukemia (ALL) [144]. Leukemia is one of the most common forms of cancer, especially found in children for which the therapeutic options thus far available are essentially unsatisfactory being associated with severe side effects [145].

Using the RCA method, Zhang and coworkers developed a multivalent system comprising multiple copies (ca. 30–40 units) of the PTK7-binding DNA aptamer known as sgc8 (5′-TCTAACTGCTGCGCCGCCGGGAAAATACTGTACGGTTAGA-3′) [146], separated by poly(dA) linkers with 3 d(GC) repeats. To improve its anticancer efficacy, the multivalent aptamer was then hybridized with doxorubicin (Dox)—a well-known DNA-intercalating chemotherapeutic agent—which should well interact with the d(GC) repetitions [147].

Compared to the monovalent species, the designed multivalent aptamer exhibited a sensibly improved binding affinity (*K*_d_ of 6.5 nM vs. 260 nM), more efficient cell internalization and selective cytotoxicity against PTK7-expressing T-leukemia CCRF-CEM cell line [147].

In a strategy similar to that explored by Di Giusto et al., the sgc8 aptamer was elaborated into a circular multivalent aptamer. In particular, each aptamer unit was modified with additional flanking sequences of 13 bases which, being complementary and thus able to form duplex tracts, allowed the formation of a circular bivalent sgc8 construct (cb-Sgc8) [148]. Notably, cb-sgc8 showed excellent in vitro resistance to nuclease degradation and proved to bind CCRF-CEM cells more efficiently than the monovalent aptamer (*K*_d_ of 0.30 nM vs. 0.86 nM) [148]. The cb-sgc8 aptamer also exhibited enhanced in vitro cell internalization and higher in vivo accumulation and retention in tumor masses compared to its monomeric component [148].

### 5.2. Nucleolin

Nucleolin is a multifunctional protein overexpressed on the outer membrane of tumor cells which exerts key roles in cell survival, growth, and proliferation [149,150]. One of the most promising aptamers, first entered in clinical trials for cancer therapy is AS1411 [151], a 26-mer G-rich DNA aptamer (5′-GGTGGTGGTGGTTGTGGTGGTGGTGG-3′), forming highly stable G4 structures, very resistant to nuclease degradation, which selectively targets this protein [152,153,154].

**Figure 4:**
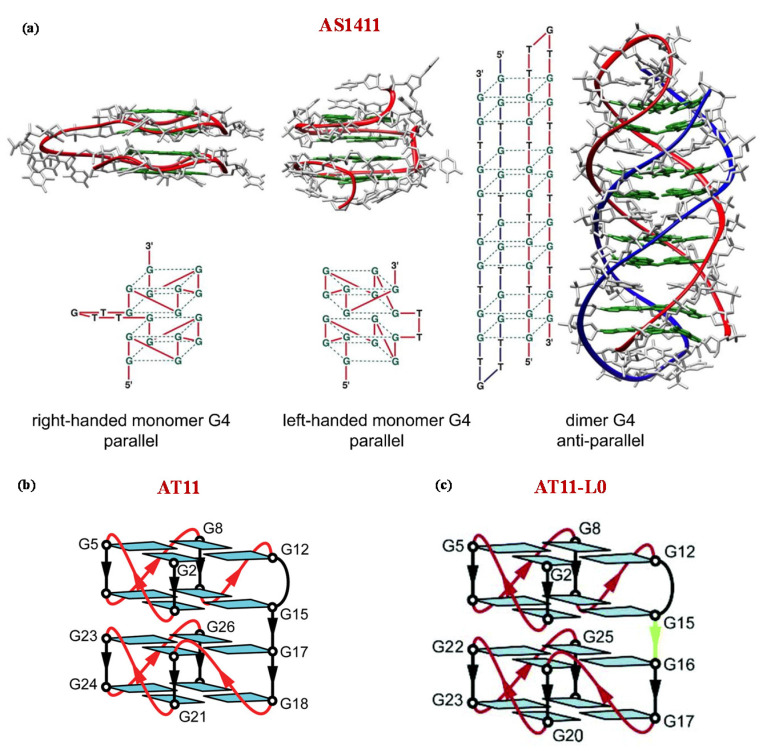
Schematic representation of the possible G-quadruplex structures of AS1411 (**a**) and NMR-derived G-quadruplex structures adopted by AT11 (**b**) and AT11-L0 (**c**) in K^+^-solutions as described by Dailey et al. [161], Do et al. [165], and Carvalho et al. [166] respectively. Figures were reproduced from refs [154,165,166] with permission.

For its intriguing properties, AS1411 has been widely used not only as a drug but also as a tumor-targeting agent. Indeed, a huge number of AS1411-based nanosystems have been successfully developed to transport and selectively deliver anticancer agents to cancer cells [154,155,156,157,158,159,160]. Detailed structural studies on this aptamer revealed its high polymorphism, and its peculiar ability to fold into multiple mono- but also dimeric G4 structures (Figure 4a) [161].

To meet the constant requirement of improved analogues with better performance, several modified AS1411 aptamers have been designed [162,163,164]. In particular, to reduce the conformational polymorphism of AS1411, Do and colleagues [165] investigated AS1411-related aptamers with single nucleotide substitution, identifying AT11 (5′-TGGTGGTGGTTGTTGTGGTGGTGGTGGT-3′), able to form a single major G4 architecture with similar antiproliferative activity as AS1411.

Detailed NMR studies proved that AT11 adopts a four-layer G4 structure containing two propeller-type parallel-stranded subunits connected through a central linker (Figure 4b) [165]. Subsequent modifications on AT11 provided the AT11-L0 aptamer (5′-TGGTGGTGGTTGTTGGGTGGTGGGGT-3′), which lacks one thymine residue between the two G4 subunits and forms a parallel-stranded G4 containing four G-quartets (Figure 4c) [165,166].

### 5.3. Vitronectin

Vitronectin (VN) is a highly glycosylated protein produced essentially in the liver and then secreted into the blood. This protein exists as a monomer, predominantly in serum, or as a multimer [167,168]. VN has a key role in wound healing and is present at elevated levels in breast cancer (BC) cells [169,170].

Thus, VN has been extensively investigated as a therapeutic target in breast cancer, the most common cancer in women representing an extremely heterogeneous malignancy with several subtypes which, growing at different rates, show distinct responses to treatments [171,172,173,174,175].

**Figure 5:**
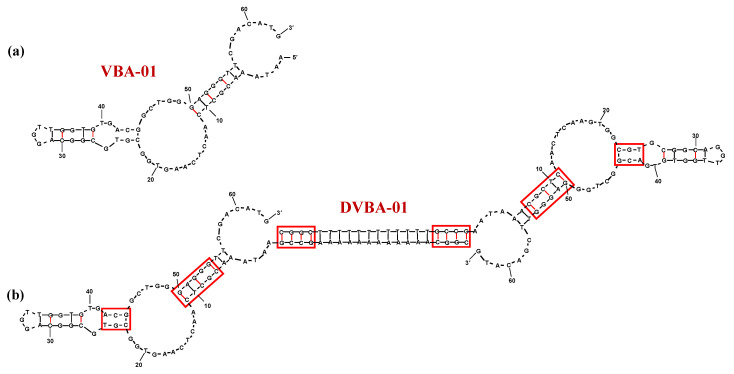
Schematic representation of the secondary structure of the monomeric and dimeric- vitronectin-targeting aptamers VBA-01 (**a**) and DVBA-01 (**b**) as predicted by the mfold software for nucleic acid folding prediction [64]. In DVBA-01 structure, red boxes indicate potential Dox-binding sites. Figures were redrawn from Stuart et al. [177].

Combining capillary electrophoresis (CE) and next-generation sequencing (NSG) for aptamer selection [176], Stuart et al. identified a novel DNA aptamer against VN, named VBA-01 (Figure 5a), with a *K*_d_ value of 405 nM (Table 1) [177]. Successively, the same authors developed DVBA-01, a dimeric aptamer obtained by extension of each VBA-01 unit with CGGC-dA_12_-GCCG or CGGC-T_12_-GCCG tracts in order to obtain a duplex connecting linker (Figure 5b). CG elongation at the ends of the poly(dA) or poly(T) tracts was specifically used since CG tracts are considered preferential binding sites for Dox. The designed dimer proved to be effective as VN-targeting compound, showing very similar binding affinity (*K*_d_ = 485 nM, Table 1) compared to the parent monomer [177].

Furthermore, using a pH-sensitive approach, DVBA-01 was then complexed with Dox and the obtained complex displayed even higher affinity for the target protein (*K*_d_ = 28 nM, Table 1) [177].

### 5.4. Prostate-Specific Membrane Antigen (PMSA)

Using a strategy very similar to that developed by Stuart and colleagues for the vitronectin aptamers [177], Boyacioglu et al. proposed a novel dimeric DNA aptamer complex bound to Dox through a pH-sensitive method [178]. This construct was specifically designed to target the prostate-specific membrane antigen (PSMA), an exopeptidase highly expressed on the apical plasma membrane of prostate cancer (PC) cells but also found in high percentage in endothelial cells from many different malignancies, including bladder, gastric, and colorectal, as well as hepatocellular, renal, breast, and ovarian cancer [179].

Notably, PSMA is present on the plasma membrane as a dimer [180], and dimeric ligands targeting this protein have shown improved activity compared to their corresponding monomers [181].

**Figure 6:**
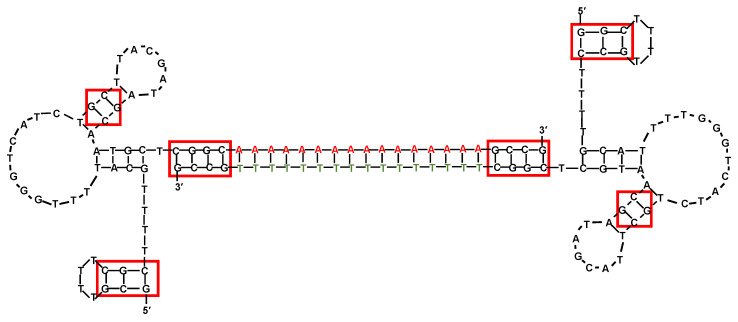
Schematic representation of the prostate-specific membrane antigen (PSMA)-targeting dimeric aptamer including two SZTI01 motifs linked by a duplex DNA “bridge” containing CG sequences appended to the ends of the dA_16_ or T_16_ bases (depicted as red and green, respectively). Red boxes indicate potential Dox-binding sites. Figure was redrawn from Boyacioglu et al. [178].

Firstly, the 48-mer DNA aptamer SZTI01, with sequence 5′-GCGTTTTCGCTTTTGCGTTTTGGGTCATCTGCTTACGATAGCAATGCT-3′, was identified using an affinity matrix system with the extracellular domain of human PSMA [178]. Then, a dimeric aptamer complex (DCA) was designed containing a duplex DNA “bridge” between the aptamer units and including the preferred binding sites for subsequent Dox incorporation (CG-rich sequences). Specifically, the authors elongated each SZTI01 motif inserting GCCG and CGGC sequences respectively to the 5′- and 3′-ends of the A_16_:T_16_ DNA duplex-forming sequence (Figure 6), thus providing a 24 base pair DNA duplex with an approximative length of 70 Å, consistent with the size of the PSMA dimer [178].

Dox-loaded dimeric aptamer proved to selectively deliver Dox to C4-2 cells, overexpressing PSMA (PMSA+) with cytotoxic effects similar to free Dox. Notably, no significant reduction of cell viability was observed on PC3 cells, which do not express PSMA, demonstrating high selectivity of the designed complex. Nevertheless, in this work, the binding affinities or IC_50_ values of these systems were not assessed [178].

### 5.5. Membrane-Bound Immunoglobulins M (mIgM)

Membrane-bound immunoglobulins M (mIgM), i.e., B-cell receptors, represent a relevant target in non-Hodgkin’s lymphomas (NHLs) [182], a neoplasm of the lymphoid tissues, which originates from B cell precursors, mature B cells, T cell precursors, and mature T cells [183].

**Figure 7:**
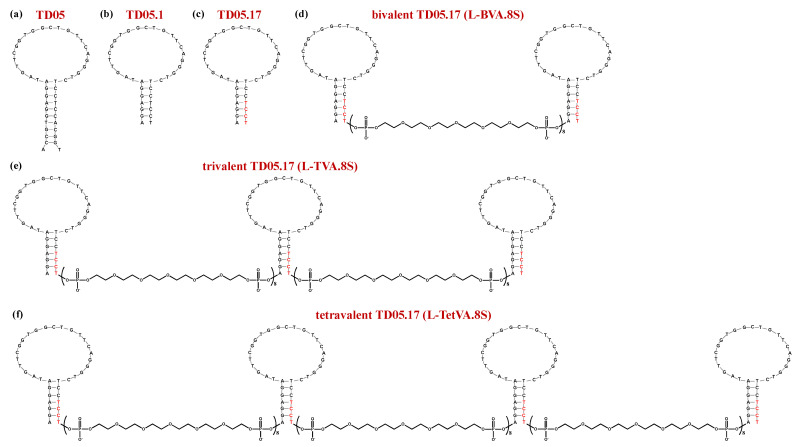
Schematic representation of the secondary structure of the monomeric and dimeric mIgM-targeting aptamers: TD05 (**a**), TD05.1 (**b**), TD05.17 (**c**), bivalent TD05.17, i.e., L-BVA.8S (**d**), trivalent TD05.17, i.e., L-TVA.8S (**e**) and tetravalent TD05.17, i.e., L-TetVA.8S (**f**). Nucleobases in red color are LNA residues. Figures were redrawn from Mallikaratchy et al. [185].

Tang and coworkers developed a high-affinity DNA aptamer (TD05, Figure 7a) reactive with Burkitt’s lymphoma cells (Ramos cells) at 4 °C [184].

Unfortunately, this aptamer was not effective in vivo because of its lack of affinity and stability at physiological temperature in human plasma. Thus, Mallikaratchy et al. produced chemically modified versions of the original 45-mer by truncation approaches and introducing locked nucleic acid (LNA) monomers in the oligonucleotide backbone of this aptamer. The best performing analogue proved to be the truncated form TD05.1 (Figure 7b)—lacking 8 nucleotides and including some nucleotide-substitutions—and its LNA derivative TD05.17 (Figure 7c), featured by the insertion of LNA residues in place of the pyrimidine nucleotides in the stem-loop region [185]. Both these derivatives exhibited dramatically enhanced binding affinity with respect to the parent sequence (Table 1) [185].

TD05.1 was then engineered into bivalent, trivalent and tetravalent constructs using HEG-based linkers (indicated as sp18) in order to increase the aptamer binding affinity at physiological temperatures [185]. Specifically, dimers of TD05.1 were prepared with spacers of 6, 8, and 12 units, corresponding to lengths of 12.6, 16.8, and 25.2 nm. All these dimers showed similar binding affinity as the parent monomer, with modest improvement only for the construct including the linker of 8 HEG units. Thus, the authors used this connecting spacer to provide trivalent (TVA.8S) and tetravalent (TetVA.8S) TD05.1-based aptamers. Compared to the monovalent sequence, both trimeric and tetrameric TD05.1 versions exhibited improved binding affinity at 37 °C (Table 1) [185].

Bi-, tri- and tetravalent aptamers were also prepared using TD05.17 (containing LNAs) and 8 HEG units as the starting monomer and the connecting linker, respectively. Trimer (L-TVA.8S) and tetramer (L-TetVA.8S) compounds were also equipped with polyethylene glycol (PEG) appendages at both extremities to improve resistance to nuclease degradation. While the bivalent form (L-BVA.8S, Figure 7d) did not show any relevant increase in binding affinity compared to the monovalent TD05.17 (Table 1), both L-TVA.8S and L-TetVA.8S (Figure 7e,f) showed a ca. 40-fold increased affinity with respect to monovalent TD05.17. In addition, these multivalent LNA-containing constructs showed ca. 2-fold enhanced binding affinity compared to those based on TD05.1 (without LNA bases), suggesting that the conformational constraints introduced by the LNA monomers highly favored the aptamer/protein interactions (Table 1) [185].

Competition and protease studies on Ramos cells also demonstrated that the multimeric aptamer TetVA.8S bound to membrane-associated human mIgM, but not to soluble IgM in plasma, allowing selective targeting of leukemia and lymphoma cells in vivo [185].

In a recent design, using a variant of cell-SELEX—known as LIgand Guided Selection (LIGS) [186,187,188]—a 79-mer aptamer, termed R1, was identified against mIgM. Since its binding affinity proved to be moderate for potential applications (*K*_d_ = 315 nM), truncated forms were then evolved [189,190]. These efforts provided two shorter versions: a 42-mer, indicated as R1.2, and a 35-mer, named R1.3, obtained as a further truncation of R1.2. Both aptamers were able to bind mIgM with an improved affinity (*K*_d_ values of 35.5 and 134 nM, respectively for R1.2 and R1.3, Table 1) and similar binding specificity compared to the parent R1 aptamer [189,190].

**Figure 8:**
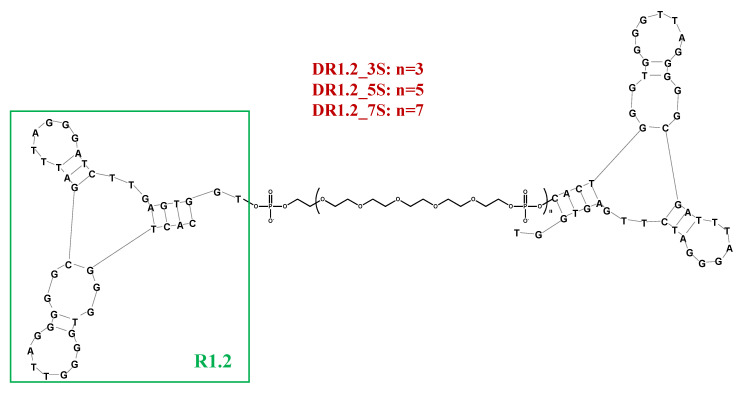
Schematic representation of the mIgM-targeting dimeric R1.2 aptamer. Figure was redrawn from Batool et al. [191].

In a subsequent work, three different homodimeric R1.2 variants were designed analyzing the impact of the linker length on the binding affinity. As previously experienced with TD05, HEG-based spacers were used. In particular, 3, 5, or 7 repeated units were inserted to join two R1.2 motifs thus providing DR1.2_3S, DR1.2_5S or DR1.2_7S (Figure 8). In all cases, dimeric constructs exhibited improved binding affinity with respect to the monovalent R1.2 aptamer especially at physiological temperatures, while at 4 °C similar or slightly improved *K*_d_ values were found (Table 1) [191].

### 5.6. T Cell Receptor Cluster of Differentiation 3 (TCR-CD3)

The T cell receptor cluster of differentiation 3 (TCR-CD3) complex, expressed on human T cells and composed from multiple domains, plays a significant role in immune cell activation [192]. Immunotherapeutic strategies, involving direct targeting of T cells in different pathologies including cancer, have completely revolutionized drug development approaches [193].

**Figure 9:**
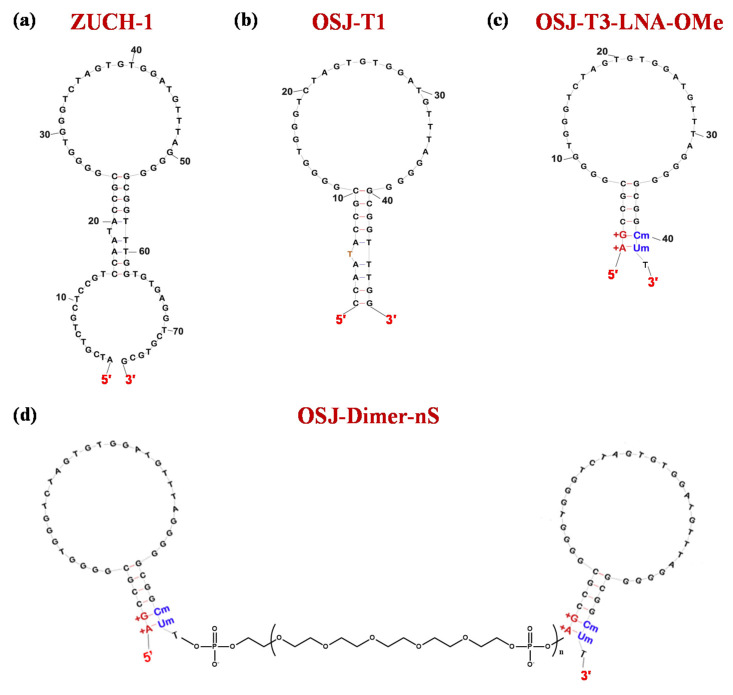
Schematic representation of the secondary structure of the monomeric and dimeric TCR-CD3-targeting aptamers: ZUCH-1 (**a**), OSJ-T1 (**b**), OSJ-T3_LNA-OMe with LNA and 2′-OMe RNA residues (**c**), bivalent OSJ-T3_LNA-OMe (**d**). LNA and 2′-OMe RNA nucleobases are marked in red and blue, respectively. Figures were adapted from Freage et al. [195] with permission.

Using LIGS approaches, aptamers able to specifically recognize the TCR-CD3 complex were identified. Among these, the 76-mer ZUCH-1 aptamer (Figure 9a) proved to be the optimal one, showing a *K*_d_ value of 3.0 nM (Table 1) [194].

To further enhance the aptamer affinity, in subsequent optimization efforts, Freage et al. designed shorter ZUCH-1 variants and then engineered suitable dimeric derivatives of the most promising truncated versions. In detail, three shorter aptamers, named OSJ-T1, OSJ-T2, and OSJ-T3—composed of 48, 49, and 39 nucleotides, respectively—were designed. These ODNs exhibited similar binding affinity compared to the parent aptamer, with OSJ-T3 (Figure 9b) as the best analogue in the series showing a *K*_d_ value of 2.1 nM (Table 1) [195].

Further modifications of OSJ-T3 provided OSJ-T3-LNA-OMe (Figure 9c), with the 1st and 2nd nucleotides from the 5′-end replaced with LNA residues, and the 40th and 41st nucleotides from the 3′-extremity substituted by 2′-OMe RNA monomers. The double modification in the original aptamer sequence proved to be very effective in terms of binding affinity, providing a *K*_d_ value of 1.7 nM (Table 1) [195].

Thus, OSJ-T3-LNA-OMe was dimerized by using from 2 (2S) to 8 (8S) repeated units of the HEG-based phosphoramidite 18 spacer (S). Three of the four designed dimers (Figure 9d) exhibited improved binding affinity compared to their monomeric counterpart. The only exception was the longer dimer OSJ-dimer-8S, which showed no improvement in binding ability as a consequence of the dimerization (Table 1) [195].

### 5.7. Vascular Endothelial Growth Factor (VEGF)

The Vascular Endothelial Growth Factor (VEGF) family is composed by different cytokine proteins involved in both vasculogenesis and angiogenesis processes [196,197,198,199,200,201,202]. VEGF-A, or simply VEGF, is the most important member of this family being able to modulate the proliferation, migration and formation of endothelial cells [203,204]. Its most abundant isoforms are VEGF_165_ and VEGF_121_ [205,206,207], which share a common receptor-binding domain (RBD) and play fundamental roles in pathological angiogenesis and vascularization of a large variety of solid tumors [208]. VEGF_165_ has also a heparin-binding domain (HBD), making this isoform more relevant than VEGF_121_ from a biological point of view [199,205,206,207].

Due to the high clinical importance of VEGF, a number of anti-VEGF nucleic acid-based aptamers has been identified by SELEX and also suitably engineered to produce homodimeric or multimeric forms [209], also taking into account the homodimeric nature of VEGF_165_ [200,201].

**Figure 10:**
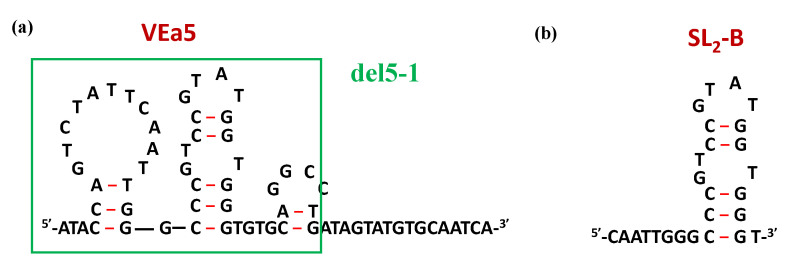
Schematic representation of the secondary structures of VEa5 (**a**) and its truncated aptamers del5-1 (green box) and SL_2_-B (**b**), as predicted by the mfold software for nucleic acid folding prediction [64]. Figures were redrawn from Hasegawa et al. [211] and Kaur et al. [212], respectively.

Through SELEX procedures, Hasegawa et al. identified a 66-mer aptamer, named VEa5 (Figure 10a), able to form a stem-loop structure and showing a *K*_d_ value of 130 nM vs. VEGF_165_ (Table 1) [210,211]. In order to obtain derivatives with increased affinity, VEa5 was subjected to successive optimization through different modifications, especially truncation of the original sequence. The same research group proposed a shorter version, i.e., del5-1 (Figure 10a) [112], while Kaur and colleagues developed the analogue known as SL_2_-B (Figure 10b) [212]. The latter compound showed a noteworthy enhancement of the binding affinity with a K_d_ value of 0.5 nM, i.e., 260-fold lower than that found for the parent aptamer (Table 1) [212].

Both the original VEa5 and its truncated forms del5-1 and SL_2_-B aptamers were then used as building blocks in the construction of suitable homodimeric species containing poly(T) linkers of different length or no linker [112,213]. In all cases, the best dimeric versions in terms of binding affinity were those obtained by direct connection of the aptamer units, not containing any spacer in between (Table 1) [112,213].

Using a post-SELEX optimization method, i.e., in silico maturation (ISM) approach, Fukaya and coworkers identified the 2G19 aptamer, a 58-mer with VEGF_165_ binding affinity in the low nanomolar range (Table 1) [214]. In the same work, the authors also designed a 2G19-based bivalent construct with a ca. 26-fold improved binding affinity with respect to its monomeric counterpart (Table 1) [214].

In detail, 2G19 comprises two different stem-loop (*sl*) regions known as *sl2* and *sl5*. The replacement of *sl2* with a *sl5* portion provided a bivalent construct with identical stem loop moieties, known as bivalent SL5. The addition of a third *sl5* domain at the 3′ end of the bivalent construct provides a trivalent species with highly improved binding affinity in comparison to the starting 2G19 aptamer (Table 1) [214].

In a more recent design, Manochehry and colleagues performed a multi-stream selection strategy, a variant of the classical SELEX procedure in which the concentration of the target protein was varied. Within this approach, they identified novel VEGF_165_-binding DNA aptamers including H4 with a *K*_d_ value of 4 nM [215]. Then, the same research group engineered a H4-based bivalent species by using an unusual, very long poly(T) spacer of 100 residues. The obtained derivative exhibited a ca. 3-fold improved binding affinity compared to the corresponding monovalent sequence (Table 1) [215].

Starting from the 33t aptamer identified by Gold and Janjic [216], Potty et al. evaluated different derivatives obtained by truncation or elongation of the original sequence leading to the +5′G+3′C analogue [217]. This aptamer was then extensively studied by Manochehry and coworkers, who built homodimeric constructs connecting two +5′G+3′C motifs with poly(T) linkers of 20 or 60 units. Both homodimers showed slightly improved binding affinity compared to their monovalent counterpart, with no significant difference associated with the linker length (Table 1) [213].

Besides DNA-based aptamers with stem-loop structures, also high affinity G-rich oligonucleotides were fished out against VEGF_165_ by SELEX [209].

In this context, Nonaka et al. selected the G-rich aptamer Vap7 able to bind both VEGF_121_ and VEGF_165_ isoforms of VEGF-A with high affinity (*K*_d_ values of 1.0 and 20 nM, respectively, Table 1) [218]. Successively, the same research group developed a truncated form, named V7t1, containing only the bases presumably involved in the G4 structure formation. Compared to the starting Vap7, V7t1 exhibited higher affinity for VEGF_165_ (*K*_d_ = 1.4 vs. 20 nM; Table 1) associated with comparable affinity for VEGF_121_ (*K*_d_ = 1.1 nM, Table 1) [218]. V7t1 also showed remarkable antiproliferative activity on several cancer cell lines [219]. However, its marked structural polymorphism in K^+^-containing solutions [220], stimulated the search of improved derivatives, such as the 3R02 aptamer (Table 1), identified by an in silico maturation approach [221]. In order to elucidate the bioactive conformation of V7t1, Moccia and coworkers recently investigated the conformational behavior of this G-rich oligomer in a Na^+^-rich buffer, mimicking the saline composition of the extracellular environment in which VEGF targeting should occur [222]. In the tested conditions, V7t1 exhibited a different structuring capability dependent on the sample preparation procedure. Indeed, V7t1 samples not subjected to annealing (i.e., directly dissolved in the selected buffer solution without any prior thermal treatment) folded in solution giving mainly dimeric parallel G4 structures, accompanied by low amounts of monomeric G4 forms. In contrast, when subjected to annealing procedures, V7t1 significantly rearranged to give exclusively monomeric G4 structures of mixed topologies [222].

Evidence of the coexistence of both monomeric and dimeric G4 forms has been obtained by native polyacrylamide gel electrophoresis (PAGE), size exclusion chromatography (SE-HPLC) and dynamic light scattering (DLS) experiments, which represent a very useful combination of techniques to verify the formation of multimers or aggregates in solution [164,223,224]. Remarkably, electrophoretic mobility shift assay (EMSA) experiments unambiguously demonstrated that only the dimeric species formed in the not-annealed V7t1 samples efficiently bound VEGF_165_, whereas the monomeric G4 species did not bind the protein under the same explored conditions [222], thus demonstrating a marked preference of the protein for dimeric G4 structures even when monomolecular forms are concomitantly present [222].

Following these results, the same research group also explored a focused set of three homodimeric V7t1 covalent dimers [225]. In detail, a poly(T) linker of 7 nucleotides, a HEG- or a TEG-based spacer were used, providing the derivatives named bisV7t1T7, bisV7t1HEG2 and bisV7t1TEG2D, respectively. In the latter analogue, i.e., bisV7t1TEG2D, the V7t1 units were connected through the 3′-extremities of each monomeric sequence, thus imposing a 3′-3′ inversion of polarity in the dimeric construct [225].

Compared to the starting V7t1 aptamer, all the covalent dimeric forms showed a slightly lower binding ability to the target protein but similar, if not slightly higher, antiproliferative activity on human breast adenocarcinoma MCF-7 cells. The species with the highest VEGF_165_ binding affinity was bisV7t1TEG2D, in which the inversion of polarity probably promotes a peculiar spatial arrangement, which is preferred by the protein [225].

Also the V7t1 analogue 3R02 was used to build a homodimeric derivative with a poly(T) spacer of 10 residues, which showed ca. 10-fold higher affinity towards VEGF_165_ than the corresponding monovalent form (Table 1) [221]. 

A very interesting VEGF-targeting heterodimeric variants was proposed by Nonaka and coworkers, who connected a DNA-forming steam loop structure with a G4-forming sequence. 

In detail, del5-1 and V7t1 were joined by a poly(T) spacer of 10 bases providing a bivalent construct with a noteworthy improvement in binding affinity with respect to each monomeric component (Table 1) [218].

### 5.8. Hepatocyte Growth Factor Receptor (HGFR)

Hepatocyte growth factor (HGF) regulates cell growth, motility and proliferation upon binding to the cell surface proto-oncogenic c-Met receptor, also called hepatocyte growth factor receptor (HGFR) [226,227,228]. In detail, activation of c-Met through dimerization triggers different signaling cascades involved in angiogenesis, cancer development and metastasis [229,230,231].

As a c-Met binding molecule, the 50-mer DNA aptamer CLN0003_SL1 (or simply SL1) was identified, showing a *K*_d_ of 123 nM (Table 1) in SNU-5 cells (c-Met+) [232].

Then, the same research group engineered several dimeric forms of SL1. The most promising analogue was a 100-mer DNA aptamer composed of two different 50-mer units, in which one is SL1 (Table 1). In cell-based experiments, this dimer (named ss-0) proved to induce c-Met activation, also reproducing HGF-induced cellular behaviors. The binding affinity constants in comparison with the starting monomers were not reported in this work [233].

## 6. Conclusions

To mediate communication between proteins, small molecules and cells, biological systems often take advantage of multivalency, in which many and cooperative low affinity interactions lead to robust and high affinity binding events [234,235].

Since aptamers isolated by SELEX do not always show the desired affinities, refinement of their properties is often necessary. Among the different post-SELEX optimization strategies, such as truncation, and chemical modification, dimerization/multimerization proved to be a very powerful and effective approach to improve aptamer binding affinity and therapeutic efficacy [50,51].

A key role in the design of dimeric or multivalent constructs is played by the connecting linker joining two or more aptamer motifs, whose length, chemical nature, and flexibility strongly influence aptamer-protein interaction. 

In this context, it could be noteworthy to underline the analogy with the connecting part of an aptazyme whose design has to mediate flexibility and stability in order to allow the proper transmission of the ligand-aptamer-signal to the catalytic nucleic acid core through a conformational change [236].

In dimeric/multimeric aptamer design, the simplest approach to link two or more aptamer sequences is represented by a simple end-to-end covalent connection, not requiring linkers to control the distance between each individual functional unit. In turn, the presence of linkers can be very effective to obtain optimized multimeric aptamers. The most simple and exploited linkers are based on poly(dA) or poly(T) sequences of variable length, which usually contain 15–20 nucleotides.

As further valuable nucleotide-based linkers, elements capable of forming well-defined secondary structures are also used. Typically, a duplex-like bridge is formed between each aptamer unit allowing their proper positioning and correct activity. Also linkers of non-nucleotidic nature, such as PEG-based ones, are frequently used for their flexibility and can be also added as terminal appendages of the aptamers to improve their enzymatic resistance to nuclease degradation.

Notably, the binding affinity or therapeutic activity of multivalent aptamers are not proportional to the number of connected monomers. Indeed, not always these properties improve as the number of single entities increases, essentially for the steric hindrance or high molecular weight of the obtained construct.

So specific features of the connecting linker have to be often defined by a trial-and-error approach, especially when high-resolution structural data (NMR or crystallographic) for the aptamer/protein complex formation are not available.

In order to improve the cooperative binding of the aptamer units to a selected protein target, there are no simple rules on the length or physico-chemical properties of the connecting linker. Indeed, even using linkers with similar properties, different results have been obtained with different aptamers.

However, the spacer has to be sufficiently long so to allow the correct aptamer structuring and should span the effective distance between the different protein binding sites.

Besides its length, also the linker orientation is a crucial factor to be considered. In fact, the linker is responsible for the spatial presentation of one aptamer unit with respect to the other one. To allow the correct orientation of each aptamer motif vs. the protein binding site, the linker inserted in the dimeric/multimeric aptamer has to be sufficiently flexible to avoid constraints, that could affect the aptamer ability to recognize and thus interact with the protein binding site.

However, bivalent and multivalent aptamers are very promising and versatile tools with potential applications as anticoagulants, anti-inflammatory, antiviral, and anticancer therapeutics. The area of these therapeutic applications and the set of newly designed highly effective multivalent aptamers will certainly be enriched in the foreseeable future.

## Figures and Tables

**Table 1:** Overview of the here discussed multivalent aptamers developed for different diseases. The specific linker between different aptamer motifs is highlighted in bold. For dimeric and multimeric constructs, the enhancement in binding affinity or activity is also reported. In LD201* aptamer, the underlined sequence is the one conserved from the original LD201 aptamer and the nucleobases in italics are those removed in the design of mΔ1. In the sequence of TD05.17 aptamer, locked nucleic acid (LNA) residues are highlighted in red. In OSJ-T3-LNA-OMe sequence, LNA and 2′-OMe RNA nucleobases are marked in red and blue, respectively. In VEa5 and Vap7 aptamers, the sequence derived from the primer regions are shown in italic lower case letters.

**Anti-inflammatory aptamers**							
**Name**	**Sequence (5′** **→3** **′)**			**Features**			**Ref.**
LD201	CAAGGTAACCAGTACAAGGTGCTAAACGTAATGGCTTCG			*K*_d_: 1.8 nM			[59]
LD174	CATTCACCATGGCCCCTTCCTACGTATGTTCTGCGGGTG			*K*_d_: 5.5 nM			
LD196	TGGCGGTACGGGCCGTGCACCCACTTACCTGGGAAGTGA			*K*_d_: 3.1 nM			
LD201*	GC*GGTAAC*CAGTACAAGGTGCTAAACGTAATGGC*GC*			IC_50_: 8 nM		T_m_: 48.7 °C	[61]
mΔ1	GCCAGTACAAGGTGCTAAACGTAATGGC			IC_50_: 10.6 nM		T_m_: 55.7 °C	
dΔ1-A3	mΔ1-**AAA**-mΔ1			IC_50_: 12 nM; **No enhancement vs. mΔ1**			
dΔ1-A9	mΔ1-**AAAAAAAAA**-mΔ1			IC_50_: 0.3 nM; **ca. 35-fold enhancement vs. mΔ1**			
dΔ1-A15	mΔ1-**AAAAAAAAAAAAAAA**-mΔ1			IC_50_: 1.6 nM; **ca. 7-fold enhancement vs. mΔ1**			
dΔ1-A20	mΔ1-**AAAAAAAAAAAAAAAAAAAA**-mΔ1			IC_50_: 1.6 nM; **ca. 7-fold enhancement vs. mΔ1**			
tΔ1-A9	mΔ1-**AAAAAAAAA**-mΔ1-**AAAAAAAAA**-mΔ1			IC_50_: 0.8 nM; **ca. 13-fold enhancement vs. mΔ1**			
**Antiviral aptamers**							
**Name**	**Sequence (5′** **→3** **′)**			**Features**			**Ref.**
1	OH-(CGGAGG)_4_-TEL			EC_50_: > 5			[78]
2	O-DMT-(CGGAGG)_4_-TEL			EC_50_: 0.54			
3	O-pGlc-(CGGAGG)_4_-TEL			EC_50_: > 5			
**Anticoagulant aptamers**							
**Name**	**Sequence (5′** **→3** **′)**		**Features**				**Ref.**
TBA15	GGTTGGTGTGGTTGG		T_m_: 33 °C		PT: 25.7 s;		[109]
AA	5′-TBA-**A-3′-3′-A**-TBA-5′		T_m_: 37 °C		PT: 32.6 s; **ca. 1.3-fold enhancement vs. TBA**		
TT	5′-TBA-**T-3′-3′-T**-TBA-5′		T_m_: 33 °C		PT: 29.6 s; **ca. 1.2-fold enhancement vs. TBA**		
gly	5′-TBA-**3′-gly-3′**-TBA-5′		T_m_: 35 °C		**No enhancement vs. TBA**		
AglyA	5′-TBA-**A-3′-gly-3′-A**-TBA-5′		T_m_: 35 °C		PT: 30.5 s; **ca. 1.2-fold enhancement vs. TBA**		
TglyT	5′-TBA-**T-3′-gly-3′-T**-TBA-5′		T_m_: 29 °C		**No activity**		
TBA	GGTTGGTGTGGTTGG		*K*_d_: 7.10 nM				[111]
TBA29	AGTCCGTGTAGGGCAGGTTGGGGTGACT		*K*_d_: 2.40 nM				
HD1-22	TBA15-**A_15_**-TBA29		*K*_d_: 0.65 nM; **ca. 11-fold enhancement vs. TBA15 and 4-fold vs. TBA29**				
Linker 0	TBA15-TBA29		*K*_d_: 0.14 nM; **ca. 144-fold enhancement vs. TBA15 and 25-fold vs. TBA29**				[112]
Linker 5	TBA-**T_5_**-TBA29		*K*_d_: 0.06 nM; **ca. 337-fold enhancement vs. TBA15 and 58-fold vs. TBA29**				
Linker 10	TBA-**T_10_**-TBA29		*K*_d_: 0.74 nM; **ca. 27-fold enhancement vs. TBA15 and 5-fold vs. TBA29**				
Linker 20	TBA-**T_20_**-TBA29		*K*_d_: 0.35 nM; **ca. 58-fold enhancement vs. TBA15 and 10-fold vs. TBA29**				
A1(12nm)A2	TBA29-**(spacer 18)_5_**-TBA15		*K*_d_: 0.3 nM; **ca. 13-fold enhancement vs. TBA15 and 10-fold vs. TBA29**				[113]
A1(24nm)A2	TBA29-**(spacer 18)_10_**-TBA15		*K*_d_: 0.06 nM; **ca. 67-fold enhancement vs. TBA15 and 48-fold vs. TBA29**				
A2(24nm)A1	TBA15-**(spacer 18)_10_**-TBA29		*K*_d_: 0.03 nM; **ca. 133-fold enhancement vs. TBA15 and 97-fold vs. TBA29**				
RNV216A	TBA15/^i^T		TCT: 27 s;				[114]
RNV219	TBA29/ ^i^T		TCT: 19 s;				
RNV220	RNV216A-**TEG**-RNV219		TCT: 40 s; **ca. 1.5-fold enhancement vs. RNV216A and 2-fold vs. RNV219**				
RNV220-T	RNV216A-**TTTT**-RNV219		TCT:30 s; **ca. 1.1-fold enhancement vs. RNV216A and 1.6-fold vs. RNV219**				
TBV-08	AGCAGCACAGAGGTCAGATG-TBA15-**TGAGACCTTGCATGCGACTTGGTGAGCACGTGAGA**-TBA29-CCTATGCGTGCTACCGTGAA		*K*_d_: 8.1 pM; **ca. 308-fold enhancement vs. TBA15 and 185-fold vs. TBA29**				[115]
16T	TBA15-T_16_-TBA29		*K*_d_: 120 pM; **ca. 21-fold enhancement vs. TBA15 and 13-fold vs. TBA29**				
Bi-4S	TBA27-**(S)_4_**-TBA15		2903 cps/sec; **No enhancement**				[116]
Bi-6S	TBA27-**(S)_6_**-TBA15		97 cps/sec; **ca. 11-fold enhancement vs. TBA15**				
Bi-8S	TBA27-**(S)_8_**-TBA15		63 cps/sec; **ca. 17-fold enhancement vs. TBA15**				
Bi-10S	TBA27-**(S)_10_**-TBA15		346 cps/sec; **ca. 3-fold enhancement vs. TBA15**				
**Anticancer aptamers**							
**Name**		**Sequence (5′→3** **′)**		**Features**			**Ref.**
VBA-01		AATAAACGCTCAACTCAAGTGGCGTGCGGCAGGTTGGTGTGACGGCTGGGAGGGTTCGACATG		*K*_d_: 405 nM			[177]
DVBA-01		VBA-01-**duplexDNA**-VBA-01		*K*_d_: 485 nM: **no enhancement**			
DVBA/Dox		DVBA-01 + Dox		*K*_d_: 28 nM; **ca. 14-fold enhancement vs. VBA-01**			
TD05		ACCGTGGAGGATAGTTCGGTGGCTGTTCAGGGTCTCCTCCACGGT		*K*_d_: 359 nM at 4 °C			[185]
TD05.1		AGGAGGATAGTTCGGTGGCTGTTCAGGGTCTCCTCCT		*K*_d_: 53 nM at 4 °C; *K*_d_ > 10,000 nM at 37 °C			
TD05.17		AGGAGGATAGTTCGGTGGCTGTTCAGGGTCTCC**TCCT**		*K*_d_: 43 nM at 4 °C: *K*_d_ > 10,000 nM at 37 °C			
TVA.8S		TD05.1-**(sp18)_8_**-TD05.1-**(sp18)_8_**-TD05.1		*K*_d_: 490 nM at 37 °C; **ca. 20-fold enhancement vs. TD05.1**			
TetVA.8S		TD05.1-**(sp18)_8_**-TD05.1-**(sp18)_8_**-TD05.1-**(sp18)_8_**-TD05.1		*K*_d_: 425 nM at 37 °C; **ca. 24-fold enhancement vs. TD05.1**			
L-BVA.8S		sp18-TD05.17-**(sp18)8**-TD05.17-sp18		*K*_d_: 6222 nM at 37 °C; **ca. 1.6-fold enhancement vs. TD05.17**			
L-TVA.8S		sp18-TD05.17-**(sp18)_8_**-TD05.17-**(sp18)_8_**-TD05.17-sp18		*K*_d_: 256 nM at 37 °C; **ca. 40-fold enhancement vs. TD05.17**			
L-TetVA.8S		sp18-TD05.17-**(sp18)_8_**-TD05.17-**(sp18)_8_**-TD05.17-**(sp18)_8_**-TD05.17-sp18		*K*_d_: 272 nM at 37 °C; **ca. 36-fold enhancement vs. TD05.17**			
R1.2		CACTGGGTGGGGTTAGCGGGCGATTTAGGGATCTTGAGTGGT		*K*_d_: 35.5 nM at 4 °C; *K*_d_: 65.6 nM at 37 °C			[189]
R1.3		CACTGGGTGGGGTTAGCGGGCGATTTAGGGATCTT		*K*_d_: 134 nM at 4 °C			
DR1.2_3S		R1.2-**(spacer)_3_**-R1.2		*K*_d_: 55.8 nM at 4 °C: **no enhancement** *K*_d_: 11.4 nM at 37 °C; **ca. 6-fold enhancement vs. R1.2**			[191]
DR1.2_5S		R1.2-**(spacer)_5_**-R1.2		*K*_d_: 31.7 nM at 4 °C; **ca. 1.1-fold enhancement vs. R1.2** *K*_d_: 20.8 nM at 37 °C; **ca. 3.1-fold enhancement vs. R1.2**			
DR1.2_7S		R1.2-**(spacer)_7_**-R1.2		*K*_d_: 23.9 nM at 4 °C; **ca. 1.5-fold enhancement vs. R1.2** *K*_d_: 48.6 nM at 37 °C; **ca. 1.3-fold enhancement vs. R1.2**			
ZUCH-1		ATCGTCTGCTCCGTCCAATACCGCGGGGTGGGTCTAGTGTGGATGTTTAGGGGGCGGTTTGGTGTGAGGTCGCGCG		*K*_d_: 3 nM			[194]
OSJ-T1		CCAATACCGCGGGGTGGGTCTAGTGTGGATGTTTAGGGGGCGGTTTGG		*K*_d_: 2.3 nM			[195]
OSJ-T2		CCAATACCGCGGGGTGGGTCTAGTGTGGATGTTTAGGGGGCGGTATTGG		*K*_d_: 2.7 nM			
OSJ-T3		GCCGCGGGGTGGGTCTAGTGTGGATGTTTAGGGGGCGGC		*K*_d_: 2.1 nM			
OSJ-T3-LNA-OMe		**AG**CCGCGGGGTGGGTCTAGTGTGGATGTTTAGGGGGCGG**CU**		*K*_d_: 1.7 nM			
OSJ-dimer-2S		OSJ-T3-LNA-OMe-**(S)_2_**-OSJ-T3-LNA-OMe		*K*_d_: 0.5 nM; **ca. 3.4-fold enhancement vs. OSJ-T3-LNA-OMe**			
OSJ-dimer-4S		OSJ-T3-LNA-OMe-**(S)_4_**-OSJ-T3-LNA-OMe		*K*_d_: 0.3 nM; **ca. 5.7-fold enhancement vs. OSJ-T3-LNA-OMe**			
OSJ-dimer-6S		OSJ-T3-LNA-OMe-**(S)_6_**-OSJ-T3-LNA-OMe		*K*_d_: 0.4 nM; **ca. 4.3-fold enhancement vs. OSJ-T3-LNA-OMe**			
OSJ-dimer-8S		OSJ-T3-LNA-OMe-**(S)_8_**-OSJ-T3-LNA-OMe		*K*_d_: 1.7 nM: **no enhancement**			
VEa5		*ATACCAGTCTATTCAATT*GGGCCCGTCCGTATGGTGGGTGTGCTGGC*AGATAGTATGTGCAATCA*		*K*_d_: 130 nM			[210,211]
del5-1		ATACCAGTCTATTCAATTGGGCCCGTCCGTATGGTGGGTGTGCTGGCCAG		*K*_d_: 476 nM			[112]
SL_2_-B		CAATTGGGCCCGTCCGTATGGTGGGT		*K*_d_: 37.9 nM			[213]
VEa5 homodimer		VEa5-VEa5		*K*_d_: 6.2 nM; **ca. 21-fold enhancement vs. VEa5**			[112]
del5-1 homodimer		del5-1-del5-1		*K*_d_: 17.2 nM; **ca. 28-fold enhancement vs. del5-1**			[112]
SL_2_-B homodimer		SL_2_-B-SL_2_-B		*K*_d_: 14 nM; **ca. 2.7-fold enhancement vs. SL_2_-B**			[213]
2G19		CTGGCCAGGTACCAAAAGATGATCTTGGGCCCGTCCGAATGGTGGGTGTTCTGGCCAG		*K*_d_: 52 nM			[214]
2G19 homodimer		2G19-2G19		*K*_d_: 2.0 nM; **ca. 26-fold enhancement vs. 2G19**			
bivalent SL_5_		SL_5_-SL_5_		1.9 nM; **ca. 27-fold enhancement vs. 2G19**			
trivalent SL_5_		SL_5_-SL_5_-SL_5_		0.37 nM; **ca. 141-fold enhancement vs. 2G19**			
H4		TTACGTCAAGGTGTCACTCCCTAGGGGTCCAGGCGAAGCTTAGTAGGGGTGTCCCCTCCCAGAAGCATCTCTTTGGCGTG		*K*_d_: 4 nM			[215]
H4 homodimer		H4-**T_100_**-H4		*K*_d_: 1.4 nM; **ca. 2.9-fold enhancement vs. H4**			
+5′G+3′C		GCCCGTCTTCCAGACAAGAGTGCAGGG C		*K*_d_: 9.9 nM			[213]
+5′G+3′C homodimer		+5′G+3′C-**T_20_**-+5′G+3′C		*K*_d_: 5.5 nM; **ca. 1.8-fold enhancement vs. +5′G+3′C**			
+5′G+3′C homodimer		+5′G+3′C-**T_60_**-+5′G+3′C		*K*_d_: 7.0 nM; **ca. 1.4-fold enhancement vs. +5′G+3′C**			
Vap7		*ATACCAGTCTATTCAATT*GCACTCTGTGGGGGTGGACGGGCCGGGT*AGATAGTATGTGCAATC*		*K*_d_: 20 nM vs. VEGF_165_; *K*_d_: 1.0 nM vs. VEGF_121_			[218]
V7t1		TGTGGGGGTGGACGGGCCGGGTAGA		*K*_d_: 1.4 nM vs. VEGF_165_; *K*_d_: 1.1 nM vs. VEGF_121_			
3R02		TGTGGGGGTGGACTGGGTGGGTACC		*K*_d_: 0.3 nM vs. VEGF_165_			[221]
3R02 homodimer		3R02-T_10_-3R02		*K*_d_: 0.03 nM vs. VEGF_165_; **ca. 10-fold enhancement vs. 3R02**			
del5-1/V7t1 heterodimer		del5-1-V7t1		*K*_d_: 0.47 nM; **ca. 1000-fold enhancement vs. del5-1 and 3-fold vs. V7t1**			[218]
CLN0003_SL1 (SL1)		ATCAGGCTGGATGGTAGCTCGGTCGGGGTGGGTGGGTTGGCAAGTCTGAT		*K*_d_: 123 nM			[232]
ss-0		ATCAGGCTGGATGGTAGCTCGGTCGGGGTGGGTGGGTTGGCAAGTCTGAT−CGTGTCACGGATGGTAGCTCGGTCGGGGTGGGTGGGTTGGCAGTGACACG		n.d.			[233]

*K*_d_: Dissociation constant; T_m_: Melting temperature; DMT: 4,4′-dimethoxytrityl; pGlc: Glucosyl-4-phosphate group; TEL: tetra-end-linked; PT: Prothrombin time; gly: Glycerol residue; TCT: Thrombin clotting time; TEG: tetraethylene glycol; Cps: Scattering intensity; n.d.: Not determined.
